# Inflammation-Associated Changes in Piezo1 Expression, Mitophagy-Related Markers, and Matrix Dysregulation in an LPS-Stimulated Co-Culture Organoid System

**DOI:** 10.3390/cells15161482

**Published:** 2026-08-18

**Authors:** Kavitha Raja, Dineshwary Grace Suresh, Jamila Khalid Albeshri, Surendra Singh Rawat, Ivan James Prithishkumar, Thomas Nau, Nerissa Naidoo

**Affiliations:** 1 College of Medicine, Mohammed Bin Rashid University of Medicine and Health Sciences, Dubai Health, Building 14, Dubai Healthcare City, Dubai P.O. Box 505055, United Arab Emirates; kavitha.raja@dubaihealth.ae (K.R.); 2376471@swansea.ac.uk (D.G.S.); 202500999@students.mbru.ac.ae (J.K.A.); surendrasingh.rawat@dubaihealth.ae (S.S.R.); ivan.prithishkumar@dubaihealth.ae (I.J.P.); 2 Faculty of Medicine, Health and Life Science, Swansea University, Singleton Park, Swansea SA2 8PP, Wales, UK; 3 King’s College Hospital London Dubai, Dubai P.O. Box 2623, United Arab Emirates; thnau@hotmail.com

**Keywords:** Piezo1, organoid, osteoarthritis, mitophagy, curcumin, autophagy

## Abstract

Osteoarthritis (OA) is a progressive joint disease characterized by cartilage degeneration, chronic low-grade inflammation, and disruption of tissue homeostasis. Although the mechanosensitive ion channel Piezo1 has been implicated in OA pathogenesis, its expression may also be modulated by inflammatory stimuli independently of applied mechanical loading. This study established a scaffold-free three-dimensional co-culture organoid model comprising human bone marrow-derived mesenchymal stem cell-derived chondrocyte-like cells and M-CSF/RANKL-differentiated RAW264.7-derived osteoclast-like cells to investigate Piezo1-associated molecular responses, inflammatory signaling, and mitophagy-related markers under lipopolysaccharide (LPS)-induced inflammatory conditions. Osteoclast-like differentiation was validated in parallel monolayer cultures by tartrate-resistant acid phosphatase staining and the presence of multinucleated cells before the corresponding differentiated cultures were used for organoid generation. Histological staining, immunofluorescence, CellTiter-Glo 3D viability assay, lactate dehydrogenase cytotoxicity assay, RT-qPCR, and Western blotting were used to evaluate extracellular matrix formation and inflammatory, catabolic, and mitochondrial quality-control-associated markers. LPS stimulation increased the expression of Piezo1, HIF-1α, phosphorylated CaMKII, NLRP3, cleaved Caspase-1, and MMP13, together with alterations in mitophagy- and autophagy-associated markers. Among the evaluated compounds, curcumin produced the greatest improvement in viability relative to the LPS-treated group and was selected for subsequent molecular analyses. Curcumin treatment was associated with reduced inflammatory and catabolic marker expression and partial preservation of cartilage-associated matrix markers. These findings demonstrate inflammation-associated changes in Piezo1 expression and related molecular markers but do not establish mechanically activated Piezo1 signaling or Piezo1-dependent causality. The organoid system therefore represents an exploratory LPS-induced inflammatory model exhibiting selected OA-relevant molecular and matrix-associated features.

## 1. Introduction

Osteoarthritis (OA) is the most prevalent degenerative joint disease and a major cause of chronic pain and disability worldwide. The disease is characterized by progressive destruction of articular cartilage, remodeling of subchondral bone, synovial inflammation, and pathological changes in surrounding joint tissues, ultimately leading to impaired joint function and reduced quality of life [1,2]. Increasing evidence indicates that OA is not solely a cartilage-associated disorder but a complex whole-joint disease involving coordinated pathological alterations across cartilage, bone, and synovial tissues [3]. During OA progression, excessive inflammatory signaling and extracellular matrix (ECM) degradation disrupts tissue homeostasis, resulting in chondrocyte dysfunction, increased catabolic activity, and abnormal bone remodeling [4]. Despite the growing global burden of OA, currently available therapies mainly provide symptomatic relief and fail to effectively halt disease progression. Although several experimental studies have investigated disease-modifying approaches, few have demonstrated effective restoration of cartilage homeostasis in osteoarthritis models [5]. Therefore, identification of molecular pathways involved in chondrogenic co-culture organoid degeneration remains essential for the development of disease-modifying therapeutic strategies.

In addition to inflammatory mediators, abnormal biomechanical stress has emerged as a critical contributor to OA pathogenesis. Physiological mechanical loading is required for maintenance of cartilage integrity and cellular homeostasis; however, excessive or dysregulated mechanical stimulation activates degenerative signaling pathways within joint tissues [6]. Mechanotransduction mediated by mechanoresponsive ion channels plays a central role in converting extracellular mechanical cues into intracellular biochemical responses. Among these channels, Piezo1 has gained significant attention as a key mechanosensitive regulator involved in cartilage degeneration [7]. Piezo1 activation promotes intracellular calcium influx and has been associated with oxidative stress, inflammatory signaling, mitochondrial dysfunction, and ECM degradation in experimental models of OA [8,9]. Increased Piezo1 expression has been reported in osteoarthritic cartilage, suggesting a potential association between aberrant mechanosensitive signaling and chondrocyte degeneration during disease progression [10]. However, the downstream relationship between Piezo1-associated calcium signaling and mitochondrial metabolic dysfunction in OA remains incompletely understood. Although Piezo1 is classically recognized as a mechanically activated ion channel, inflammatory mediators may also regulate its expression and modify cellular responsiveness. Previous studies have reported changes in Piezo1 expression following inflammatory stimulation, including exposure to LPS and pro-inflammatory cytokines. However, altered Piezo1 abundance under inflammatory conditions is not equivalent to demonstration of mechanically activated channel function. Therefore, in the absence of mechanical loading, calcium imaging, electrophysiological measurements, or Piezo1-specific modulation, the present study evaluates Piezo1 as an inflammation-responsive molecular marker rather than as a direct functional measure of mechanotransduction.

Emerging evidence further demonstrates that metabolic reprogramming and mitochondrial dysfunction are closely associated with OA progression. Chondrocytes rely predominantly on glycolytic metabolism for energy production because of the hypoxic cartilage microenvironment [11]. Hypoxia-inducible factor-1α (HIF-1α) functions as a critical regulator of cellular metabolic adaptation by promoting glycolysis and maintaining chondrocyte survival under stress conditions [12]. Nevertheless, dysregulated HIF-1α signaling has been associated with abnormal metabolic activity, lactate accumulation, mitochondrial stress, and inflammatory responses in OA tissues [13]. Mitochondrial dysfunction leads to excessive reactive oxygen species (ROS) production, impaired ATP generation, and activation of pro-inflammatory pathways that accelerate cartilage degradation and chondrocyte apoptosis [14]. Previous studies involving direct Piezo1 activation have suggested a relationship between Piezo1-mediated calcium signaling and mitochondrial homeostasis [15]. In the present inflammation-based system, however, Piezo1 channel activity and intracellular calcium influx were not directly assessed; therefore, the analysis was limited to associations between Piezo1 expression and mitochondrial quality-control-related markers.

Maintenance of mitochondrial integrity is tightly regulated through mitophagy, a selective autophagic process responsible for eliminating damaged mitochondria and preserving cellular homeostasis [16]. Impairment of mitophagy results in accumulation of dysfunctional mitochondria, enhanced oxidative stress, and activation of inflammatory cascades that contribute to OA progression [17]. Key mitophagy regulators, including PTEN-induced kinase 1 (PINK1) and Parkin, are involved in mitochondrial quality control and cellular stress adaptation [18]. Disruption of these pathways has been associated with inflammasome activation, increased catabolic signaling, and extracellular matrix ECM degradation in OA chondrocytes [19]. Previous studies have suggested that Piezo1-associated calcium signaling may influence mitochondrial quality-control mechanisms and mitophagy-related processes, providing a possible relationship among Piezo1 activity, metabolic stress, and inflammatory signaling in OA [20]. However, this functional relationship was not directly examined in the present study. Nevertheless, the relationships among inflammation-associated Piezo1 expression, HIF-1α, and mitophagy-related molecular markers remain incompletely characterized.

Advanced three-dimensional (3D) culture systems have gained increasing importance for studying OA pathophysiology because conventional two-dimensional monolayer cultures fail to accurately reproduce the native microenvironment [21]. Scaffold-free organoid systems provide enhanced cell–cell interactions, endogenous ECM deposition, and spatial tissue organization that better mimic physiological joint architecture [22]. hBMSC-derived chondrocyte-like cells have been used for in vitro three-dimensional culture development because of their chondrogenic differentiation capacity and extracellular matrix-producing potential [23]. Incorporation of validated osteoclast-like cells may provide an additional bone-remodeling and inflammatory cellular component within the co-culture microenvironment [24]. These organoid systems provide controlled three-dimensional platforms for investigating cell–cell interactions, cartilage-associated matrix changes, inflammatory responses, and candidate interventions. However, their physiological and translational relevance depends on the cellular composition, inflammatory stimulus, and degree of functional validation.

Osteoarthritis is primarily characterized by sterile, low-grade, and chronic inflammation arising from mechanical stress, matrix degradation products, cellular senescence, and damage-associated molecular patterns, including high-mobility group box 1 (HMGB1). Several endogenous danger signals can activate Toll-like receptor 4 (TLR4)-dependent pathways that contribute to inflammatory and catabolic signaling in OA. In contrast, lipopolysaccharide (LPS) is an exogenous bacterial TLR4 agonist and is not a physiological initiating factor for degenerative OA. Nevertheless, LPS provides a reproducible experimental stimulus for inducing acute TLR4-dependent inflammation, inflammasome-associated responses, mitochondrial stress, and matrix-catabolic changes in vitro. Accordingly, LPS was used in the present study as a surrogate inflammatory challenge to produce selected OA-relevant molecular features rather than to reproduce the chronic, sterile, mechanically influenced, and multifactorial pathogenesis of clinical OA [25].

In parallel, naturally derived bioactive compounds and nutraceutical agents have attracted significant interest because of their anti-inflammatory, antioxidant, and mitochondrial protective properties [26]. Several nutraceutical compounds have demonstrated potential to regulate inflammatory signaling, restore mitochondrial function, and suppress cartilage catabolism, thereby supporting their therapeutic relevance in OA management [27]. However, the molecular mechanisms through which these compounds influence mechanosensitive and metabolism-associated pathways in osteoarthritis remain incompletely understood.

Therefore, the present study aimed to establish a scaffold-free 3D chondrogenic–osteoclast-like co-culture organoid system and investigate associations among LPS-induced inflammatory stress, Piezo1 expression, mitochondrial quality-control-associated markers, inflammasome-related responses, and extracellular matrix homeostasis. hBMSC-derived chondrocyte-like cells were co-cultured with M-CSF/RANKL-differentiated and TRAP-validated RAW264.7-derived osteoclast-like cells to generate a three-dimensional platform for evaluating controlled TLR4-mediated inflammatory responses. Because mechanical loading, Piezo1 channel-activity measurements, calcium imaging, and Piezo1-specific pharmacological or genetic manipulation were not performed, the study was not designed to establish mechanically activated Piezo1 signaling or Piezo1-dependent causality. We hypothesized that LPS-induced inflammatory stimulation would be associated with changes in Piezo1 and related molecular markers and that these changes would be attenuated by curcumin.

## 2. Materials and Methods

### 2.1. Ethical and Biosafety Compliance

All experimental procedures were conducted in accordance with the approved laboratory biosafety regulations and Good Laboratory Practice (GLP) guidelines of Mohammed Bin Rashid University of Medicine and Health Sciences (MBRU), Dubai Health, Dubai, United Arab Emirates. All cell lines, reagents, and biological materials used in this study were obtained from certified commercial suppliers and utilized exclusively for research purposes.

### 2.2. Cell Culture and Maintenance

hBMSCs were obtained from Cytion GmbH (Eppelheim, Germany) and cultured in Dulbecco’s Modified Eagle Medium/Nutrient Mixture F-12 (DMEM/F-12; Gibco, Thermo Fisher Scientific, Waltham, MA, USA; Cat. No. 11320033) supplemented with 10% fetal bovine serum (FBS; Gibco, Thermo Fisher Scientific; Cat. No. 16000044) and 1% penicillin– streptomycin (100×; Gibco, Thermo Fisher Scientific; Cat. No. 15140122). Cells between passages 3 and 5 were used for all experiments. RAW264.7 murine macrophages were obtained from Cytion GmbH (Eppelheim, Germany) and maintained in DMEM/F12 supplemented with 10% FBS and 1% penicillin–streptomycin under standard humidified culture conditions (37 °C, 5% CO_2_).

### 2.3. Chondrogenic Differentiation of BMSCs

hBMSCs at passages 3–5 were induced toward chondrogenic differentiation using StemPro™ Chondrogenesis Differentiation Medium (Thermo Fisher Scientific; Cat. No. A1007101) according to the manufacturer’s instructions. Cells were maintained in chondrogenic differentiation medium for 21 days, with the medium replaced every 2–3 days. Following differentiation, the cells were used for organoid generation and are described throughout the manuscript as “hBMSC-derived chondrocyte-like cells” because they were derived from hBMSCs and were not primary adult articular chondrocytes. Chondrogenic characteristics were subsequently evaluated at the organoid-characterization stage using cartilage-associated histological staining and assessment of SOX9 and COL2A1 expression. Differentiation purity was not quantified at the single-cell level, and hypertrophic differentiation markers, including COL10A1 and RUNX2, were not evaluated.

### 2.4. RAW264.7-Derived Osteoclast-like Cell Differentiation and Validation

RAW264.7 murine macrophage-like cells were seeded in standard 24-well tissue-culture plates at a density of 1.5 × 10^4^ cells per well in 500 µL of complete DMEM/F-12 containing 10% fetal bovine serum and 1% penicillin–streptomycin. Cells were allowed to adhere overnight. Osteoclast-like differentiation was induced by replacing the growth medium with differentiation medium supplemented with recombinant murine M-CSF (50 ng/mL; PeproTech, Cranbury, NJ, USA; Cat. No. 315-02) and recombinant murine RANKL (50 ng/mL; PeproTech; Cat. No. 315-11). The differentiation medium containing freshly added M-CSF and RANKL was replenished on day 3. Following 5 days of differentiation, cells were fixed with 4% paraformaldehyde and stained using a Leukocyte Acid Phosphatase TRAP Kit (Sigma-Aldrich, St. Louis, MO, USA; Cat. No. 387A-1KT) according to the manufacturer’s instructions. Osteoclast-like differentiation was supported by the presence of TRAP-positive multinucleated cells containing three or more nuclei (Supplementary Figure S2). Because functional bone-resorption activity was not assessed, the differentiated cells are described as RAW264.7-derived osteoclast-like cells rather than fully mature functional osteoclasts [24].

### 2.5. Scaffold-Free 3D Chondrogenic Co-Culture or Ganoid Generation

Following M-CSF/RANKL-mediated differentiation, RAW264.7-derived osteoclast-like cells were harvested using Accutase cell detachment solution, counted, and co-cultured with hBMSC-derived chondrocyte-like cells. Parallel monolayer cultures subjected to the same differentiation protocol were used for TRAP validation, whereas corresponding unstained differentiated cultures were harvested for organoid generation. Scaffold-free 3D co-culture organoids were generated by seeding 10,000 hBMSC-derived chondrocyte-like cells and 10,000 RAW264.7-derived osteoclast-like cells at a 1:1 ratio into ultra-low-attachment 96-well plates (Corning Inc., Corning, NY, USA; Cat. No. 7007), corresponding to 20,000 cells per organoid. The organoid culture medium consisted of DMEM/F-12 supplemented with 10% fetal bovine serum and 1% penicillin–streptomycin. Organoids were maintained for 6 days before LPS stimulation. For each experimental condition, three organoids were analyzed as technical replicates within each independent biological replicate. Scaffold-free organoid technology was selected because it is effective in mimicking native cell–cell and cell–matrix interactions observed within the chondrogenic co-culture organoid microenvironment [28,29,30].

Organoids were maintained at 37 °C and 5% CO_2_, and culture medium was replaced every 2 days. Organoid formation and maturation were monitored using bright-field microscopy and Incucyte live-cell imaging throughout the culture period.

### 2.6. Histological Characterization of Organoids

Organoids were fixed in 4% paraformaldehyde, processed, embedded, and sectioned. Hematoxylin and eosin (H&E) staining was performed to evaluate general cellular morphology and tissue organization. Alcian Blue and Toluidine Blue staining were used to assess glycosaminoglycan and proteoglycan deposition within the ECM. Safranin O–Fast Green staining was performed to evaluate cartilage-associated ECM deposition [31,32].

### 2.7. Immunofluorescence Characterization

Organoid sections were fixed, permeabilized using Triton X-100, and blocked with bovine serum albumin prior to antibody incubation. Samples were incubated overnight at 4 °C with primary antibodies against SOX9 and COL2A1. Following washing steps, fluorophore-conjugated secondary antibodies were applied and nuclei were counterstained using DAPI (Invitrogen, Thermo Fisher Scientific; Cat. No. D1306). Fluorescence images were captured using a fluorescence/confocal microscope under identical imaging settings.

SOX9 and COL2A1 immunostaining were performed to evaluate chondrogenic marker expression and cartilage-associated ECM formation [33,34]. Fluorescence intensity was quantified using ImageJ software (version 1.54, National Institutes of Health, Bethesda, MD, USA) and normalized relative to DAPI-positive nuclei.

### 2.8. Induction of LPS-Mediated Inflammatory and Catabolic Stress

Mature co-culture organoids were exposed to lipopolysaccharide (LPS) from *Escherichia coli* O111:B4 (Sigma-Aldrich, St. Louis, MO, USA; Cat. No. L2630) at 1 µg/mL for 24 h to induce an acute TLR4-mediated inflammatory and catabolic response. LPS was used as a reproducible surrogate inflammatory stimulus and not as a direct representation of the sterile, chronic, and mechanically influenced initiation of clinical OA. Therefore, the experimental system is interpreted as an LPS-induced inflammatory organoid model exhibiting selected OA-relevant molecular and matrix-associated features.

### 2.9. Candidate Compound Treatment and Dose Screening

Following LPS induction, organoids were treated with selected candidate compounds, including Curcumin (≥95%; Sigma-Aldrich; Cat. No. C1386), Rivaroxaban (MedChemExpress; Monmouth Junction, NJ, USA; Cat. No. HY-10027) and Sphingosine-1-phosphate (Cayman Chemical; Ann Arbor, MI, USA; Cat. No. 22498). Dose-dependent effects on organoid viability were evaluated using the CellTiter-Glo 3D assay. Tested concentrations included curcumin (1.8, 3.7, 7.4, and 14.7 µg/mL), rivaroxaban (0.22, 0.44, 1.09, and 2.18 µg/mL), and S1P (0.019, 0.038, 0.095, and 0.19 µg/mL). Based on the viability screening results, curcumin (7.4 µg/mL) was selected for subsequent molecular analyses. The evaluated concentrations were selected based on preliminary dose-screening experiments.

### 2.10. Experimental Groups

Organoids were divided into the following experimental groups: (1) untreated control organoids, (2) LPS-stimulated organoids, and (3) LPS-stimulated organoids treated with curcumin. Additional preliminary screening groups included LPS-stimulated organoids treated with rivaroxaban or S1P for dose optimization studies. All subsequent molecular analyses were performed using curcumin at 7.4 µg/mL.

### 2.11. Cell Viability and Cytotoxicity Assays

Organoid viability was quantified using the CellTiter-Glo^®^ 3D Cell Viability Assay (Promega, Madison, WI, USA; Cat. No. G9681) according to the manufacturer’s instructions. Luminescence intensity was normalized to untreated control organoids and expressed as percentage viability. To further assess viability and cytotoxicity, Live/Dead staining was performed using calcein-AM and ethidium homodimer-1/propidium iodide [35,36]. Viable cells exhibited green fluorescence, whereas dead cells displayed red fluorescence. Cytotoxicity was evaluated using the LDH Cytotoxicity Detection Kit (Roche Diagnostics; Basel, Switzerland; Cat. No. 11644793001) according to the manufacturer’s instructions.

### 2.12. RNA Extraction and Quantitative Real-Time PCR (RT-qPCR)

Total RNA was extracted from organoids using TRIzol™ Reagent (Invitrogen, Thermo Fisher Scientific; Cat. No. 15596026) according to the manufacturer’s instructions. RNA concentration and purity were assessed spectrophotometrically prior to cDNA synthesis. Reverse transcription was performed using the High-Capacity cDNA Reverse Transcription Kit (Applied Biosystems; Waltham, MA, USA; Cat. No. 4368814). Quantitative real-time PCR was conducted using PowerUp™ SYBR™ Green Master Mix (Applied Biosystems; Cat. No. A25742).

Given the interspecies composition of the co-culture organoids, species-specific primer assays were used to distinguish transcriptional responses associated with the human BMSC-derived chondrocyte-like and murine RAW264.7-derived osteoclast-like components. Human-specific RT-qPCR assays were used to assess *PIEZO1*, *HIF1A*, *CAMK2D*, *PINK1*, *PRKN*, *SQSTM1*, *MAP1LC3B*, *COL2A1*, and *MMP13*. Mouse-specific RT-qPCR assays were used to assess Nlrp3 and Casp1. Human target-gene expression was normalized using a human-specific GAPDH assay, whereas murine target-gene expression was normalized separately using a mouse-specific Gapdh assay. Relative gene expression was calculated using the 2^−ΔΔCq^ method, with the untreated control group used as the calibrator [37,38,39].

### 2.13. Western Blot Analysis

Total cellular protein was extracted from organoids using RIPA Lysis Buffer (Thermo Fisher Scientific; Cat. No. 89900) supplemented with Protease Inhibitor Cocktail (Thermo Fisher Scientific; Cat. No. 78430) and Phosphatase Inhibitor Cocktail (Thermo Fisher Scientific; Cat. No. 78420). Protein concentrations were determined using the Pierce™ BCA Protein Assay Kit (Thermo Fisher Scientific; Cat. No. 23225). Equal amounts of protein were separated by SDS-PAGE and transferred onto Immobilon-P PVDF membranes (MilliporeSigma; Burlington, MA, USA; Cat. No. ISEQ00010) using established Western blot procedures [40,41]. Membranes were blocked and incubated overnight at 4 °C with primary antibodies against Piezo1, HIF-1α, phosphorylated CaMKII (p-CaMKII), total CaMKII, PINK1, Parkin, LC3B, p62/SQSTM1, NLRP3, cleaved Caspase-1 p20, COL2A1, MMP13, and GAPDH. Following incubation with HRP-conjugated secondary antibodies, immunoreactive bands were visualized using SuperSignal West Pico PLUS Chemiluminescent Substrate (Thermo Fisher Scientific; Cat. No. 34580). Densitometric analysis was performed using ImageJ software [42]. Protein expression was normalized relative to GAPDH, whereas p-CaMKII levels were normalized to total CaMKII expression. LC3B-I and LC3B-II expression levels were quantified as indicators of autophagy-associated responses [43]. Original uncropped Western blot images corresponding to Figures 6–8, with molecular weight markers shown where available, are provided in Supplementary Figure S1. Detailed information regarding primary antibodies, catalogue numbers, host species, and working dilutions is provided in Supplementary Table S1.

### 2.14. Statistical Analysis

All experiments were performed independently three times using separately generated organoid cultures (n = 3 biological replicates). Within each biological replicate, three organoids per experimental condition were analyzed as technical replicates, and their measurements were averaged to obtain one value per biological replicate. Data are presented as the mean ± standard deviation (SD) of the three biological replicates. Statistical analyses were performed using GraphPad Prism version 10.2 (GraphPad Software, San Diego, CA, USA). Comparisons among groups were performed using one-way analysis of variance (ANOVA), followed by Tukey’s multiple-comparisons test [44]. A *p*-value < 0.05 was considered statistically significant. Statistical significance is indicated as * *p* < 0.05, ** *p* < 0.01, *** *p* < 0.001, and **** *p* < 0.0001. Where two comparator groups were indicated, hash symbols denote comparisons with the untreated control group, whereas asterisks denote comparisons with the LPS-treated group.

## 3. Results

### 3.1. Validation of RAW264.7-Derived Osteoclast-like Differentiation

RAW264.7 cells cultured with M-CSF (50 ng/mL) and RANKL (50 ng/mL) for 5 days demonstrated osteoclast-associated morphological changes and formation of TRAP-positive multinucleated cells containing three or more nuclei. These findings supported osteoclast-like differentiation before incorporation of the corresponding differentiated cells into the scaffold-free co-culture organoid system. Because functional bone-resorption activity was not evaluated, the differentiated cells were interpreted as osteoclast-like cells rather than fully mature functional osteoclasts. Representative TRAP-stained monolayer cultures are presented in Supplementary Figure S2.

### 3.2. Formation and Morphological Characterization of Scaffold-Free Co-Culture Organoids

**Figure 1:**
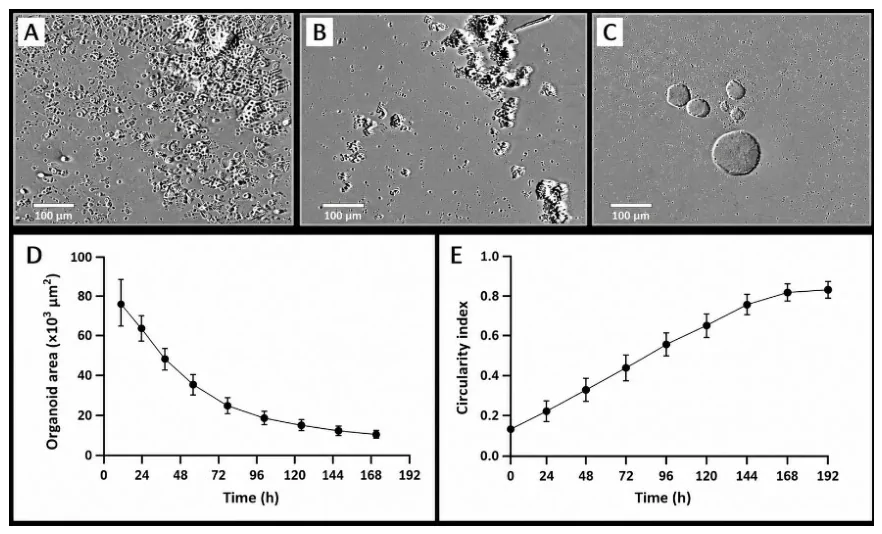
Time-course development and maturation of scaffold-free chondrogenic co-culture organoids monitored by live-cell imaging. (**A**–**C**) Representative bright-field images of scaffold-free co-culture organoid formation at 0, 72, and 144 h, respectively. Scale bar = 100 µm. (**D**) Quantitative analysis of organoid area over time. A progressive reduction in organoid area was observed throughout the culture period, reflecting cellular condensation and spheroid compaction during organoid maturation. Data are presented as mean ± SD (n = 3). (**E**) Quantitative analysis of organoid circularity over time. Circularity values increased progressively during culture, indicating the transition from irregular cellular aggregates to structurally organized and compact spheroid-like organoids. Data are presented as mean ± SD (n = 3).

Scaffold-free co-culture organoids were generated by co-culturing hBMSC-derived chondrocyte-like cells with M-CSF/RANKL-differentiated RAW264.7-derived osteoclast-like cells. Bright-field imaging demonstrated progressive self-assembly and compaction of the organoids over time. During the initial phase (Days 1–2), loosely associated multicellular aggregates with irregular morphology were observed. By Days 2–4, enhanced cellular condensation and formation of dense micro-aggregates became evident. Mature compact spherical organoids with smooth and well-defined borders were consistently observed between Days 5–8, indicating successful organoid maturation and structural stabilization (Figure 1A–E).

### 3.3. Histological Characterization of Cartilage-like Extracellular Matrix Formation

**Figure 2:**
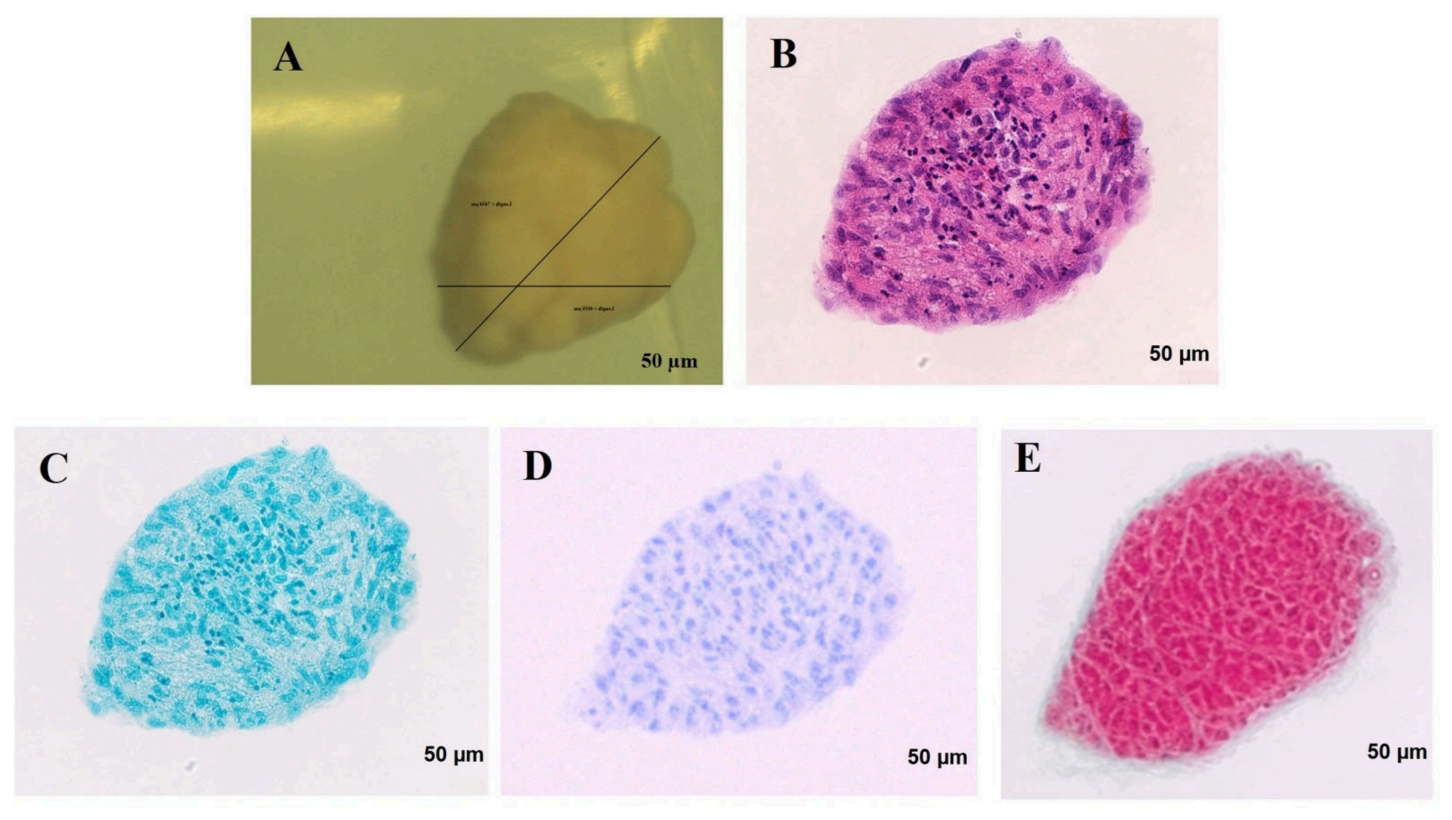
Morphological and histological characterization of scaffold-free chondrogenic organoids. (**A**) Bright-field image showing compact scaffold-free organoid formation following spontaneous cellular self-aggregation. (**B**) Hematoxylin and eosin (H&E) staining demonstrating preserved spheroid architecture and dense cellular organization. (**C**) Alcian Blue staining confirming glycosaminoglycan-rich extracellular matrix deposition within the organoid structure. (**D**) Toluidine Blue staining demonstrating proteoglycan-associated cartilage-like matrix formation. (**E**) Safranin O–Fast Green staining showing abundant proteoglycan-rich extracellular matrix deposition and chondrogenic maturation within scaffold-free organoids. Representative images were acquired under identical magnification settings. Scale bar = 50 µm.

Histological staining supported cartilage-like ECM formation within the scaffold-free organoids. H&E staining demonstrated dense cellular organization throughout the spheroid structure. Alcian Blue staining revealed glycosaminoglycan-rich ECM deposition within the ECM. Toluidine Blue staining further confirmed the presence of proteoglycan-rich matrix components, while Safranin O–Fast Green staining demonstrated strong cartilaginous ECM formation and chondrogenic maturation of the organoids. Collectively, these findings were consistent with chondrogenic differentiation, cartilage-like matrix formation and establishment of a cartilage-like microenvironment within the scaffold-free organoid system (Figure 2A–E).

### 3.4. Immunofluorescence Characterization of Chondrogenic Differentiation

**Figure 3:**
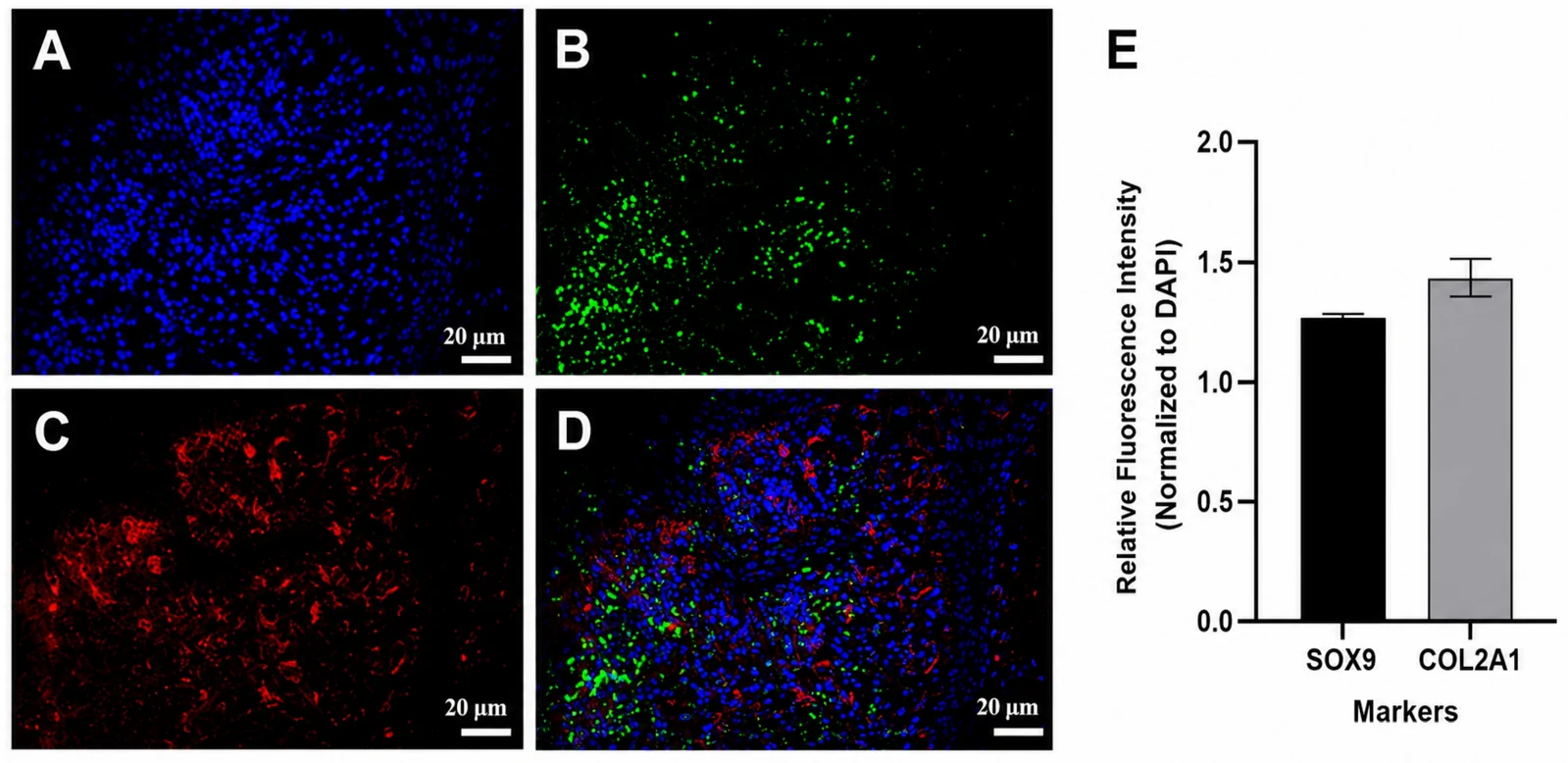
Immunofluorescence characterization of chondrogenic marker expression in scaffold-free chondrogenic co-culture organoids. Representative immunofluorescence images demonstrating the expression of key chondrogenic markers within scaffold-free chondrogenic co-culture organoids. (**A**) DAPI staining (blue) showing the distribution of cell nuclei throughout the organoid structure. (**B**) SOX9 immunostaining (green), indicating the presence of chondrogenically differentiated cells and maintenance of the chondrogenic phenotype. (**C**) Collagen type II alpha 1 (COL2A1) immunostaining (red), demonstrating the deposition of cartilage-specific extracellular matrix within the organoid. (**D**) Merged image showing the spatial localization of DAPI, SOX9, and COL2A1 signals. The positive expression of SOX9 and COL2A1 supports successful chondrogenic differentiation and cartilage-like matrix formation within the scaffold-free organoid model. Scale bar = 20 µm. (**E**) Quantitative fluorescence intensity analysis of SOX9 and COL2A1 immunofluorescence signals was performed using ImageJ and expressed as mean ± SD from three independent biological replicates (n = 3).

Immunofluorescence analysis demonstrated expression of chondrogenic markers within the organoids, together with cartilage-associated ECM formation. DAPI staining confirmed dense cellular distribution throughout the organoid structure. Strong SOX9 immunoreactivity was observed, indicating activation of chondrogenic transcriptional regulation. In addition, robust COL2A1 expression was detected within the extracellular matrix, supporting cartilage-associated matrix deposition. Merged fluorescence images demonstrated co-localization of SOX9-positive cells with COL2A1-rich extracellular regions, supporting chondrogenic maturation of the scaffold-free co-culture organoids (Figure 3A–E). Quantitative fluorescence analysis demonstrated strong SOX9 and COL2A1 signal intensity within mature organoids.

### 3.5. Dose-Dependent Effects of Nutraceutical Compounds on Organoid Viability

**Figure 4:**
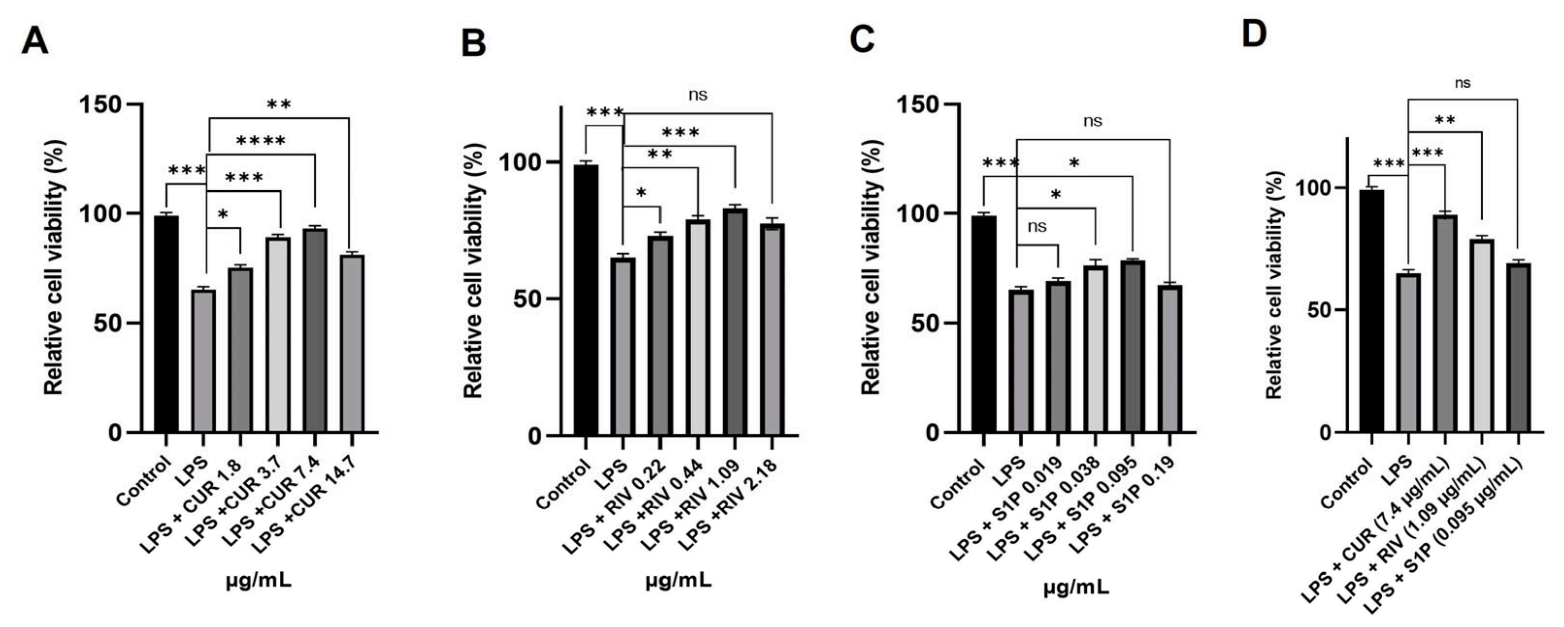
Screening of candidate compounds for the selection of optimal treatment concentrations in LPS-stimulated scaffold-free chondrogenic co-culture organoids. (**A**) Effect of increasing concentrations of curcumin (1.8, 3.7, 7.4, and 14.7 µg/mL) on organoid viability following LPS stimulation. (**B**) Effect of increasing concentrations of rivaroxaban (0.22, 0.44, 1.09, and 2.18 µg/mL) on organoid viability following LPS stimulation. (**C**) Effect of increasing concentrations of sphingosine-1-phosphate (S1P; 0.019, 0.038, 0.095, and 0.19 µg/mL) on organoid viability following LPS stimulation. (**D**) Comparative analysis of the selected optimal concentrations of curcumin (7.4 µg/mL), rivaroxaban (1.09 µg/mL), and S1P (0.095 µg/mL). LPS treatment significantly reduced organoid viability compared with the untreated control group, whereas the candidate-compound groups showed higher viability relative to the LPS-treated group in a concentration-dependent manner. Based on comparative viability screening, curcumin at 7.4 µg/mL was selected for subsequent molecular analyses. Data are presented as mean ± SD (n = 3 independent experiments). Statistical significance was determined using one-way ANOVA followed by Tukey’s multiple comparison test. * *p* < 0.05, ** *p* < 0.01, *** *p* < 0.001, and **** *p* < 0.0001. ns, not significant.

The effects of curcumin, rivaroxaban, and S1P on chondrogenic co-culture organoid viability were evaluated following LPS-induced inflammatory stimulation. LPS treatment significantly reduced organoid viability compared with untreated controls. Under LPS-stimulated conditions, curcumin, rivaroxaban, and S1P were associated with concentration-dependent differences in organoid viability relative to the LPS-treated group. Among the tested compounds, curcumin produced the greatest improvement in viability relative to the LPS-treated group and maintained organoid morphology and structural integrity. Based on the viability screening results, curcumin at 7.4 µg/mL was selected for subsequent molecular analyses (Figure 4A–D).

### 3.6. Curcumin Treatment Is Associated with Improved Viability and Reduced Cytotoxicity Under Inflammatory Conditions

Live/Dead staining demonstrated high cellular viability in untreated control organoids, characterized predominantly by green fluorescence. In contrast, LPS-treated organoids exhibited marked increases in red fluorescence, indicating elevated cell death and reduced viability under inflammatory conditions. Curcumin treatment was associated with increased viability-associated fluorescence relative to the LPS group, as demonstrated by restoration of green fluorescence and reduction of red fluorescent signals.

**Figure 5:**
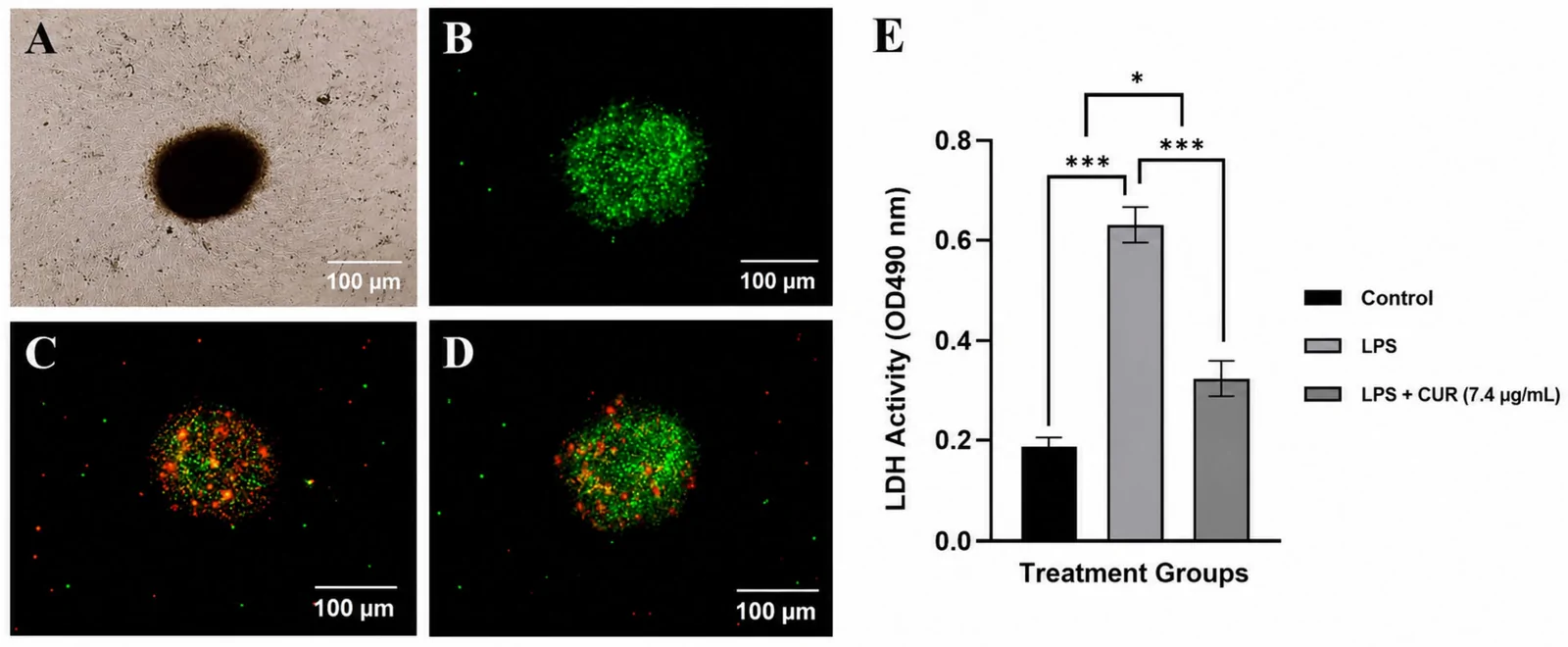
Assessment of viability and cytotoxicity in scaffold-free chondrogenic co-culture organoids. (**A**) Representative bright-field image of a scaffold-free organoid. (**B**) Calcein-AM staining (green) identifying viable cells. (**C**) Propidium iodide staining (red) identifying non-viable cells with compromised membrane integrity. (**D**) Merged fluorescence image showing the distribution of viable and non-viable cells. Scale bars = 100 µm. (**E**) Quantification of lactate dehydrogenase (LDH) release in untreated control, LPS-treated, and LPS plus curcumin-treated (7.4 µg/mL) organoids. Data are presented as mean ± SD (n = 3 independent experiments). Statistical significance was determined using one-way ANOVA followed by Tukey’s multiple-comparisons test. * *p* < 0.05 and *** *p* < 0.001.

Consistent with these observations, LDH release assay demonstrated significantly increased cytotoxicity following LPS stimulation. Curcumin treatment significantly reduced LDH release compared with LPS-treated organoids, indicating lower cytotoxicity and improved cellular integrity relative to the LPS-treated group within the chondrogenic co-culture organoids (Figure 5A–E).

### 3.7. LPS Stimulation Is Associated with Increased Piezo1 and Calcium-Responsive Molecular Marker Expression

**Figure 6:**
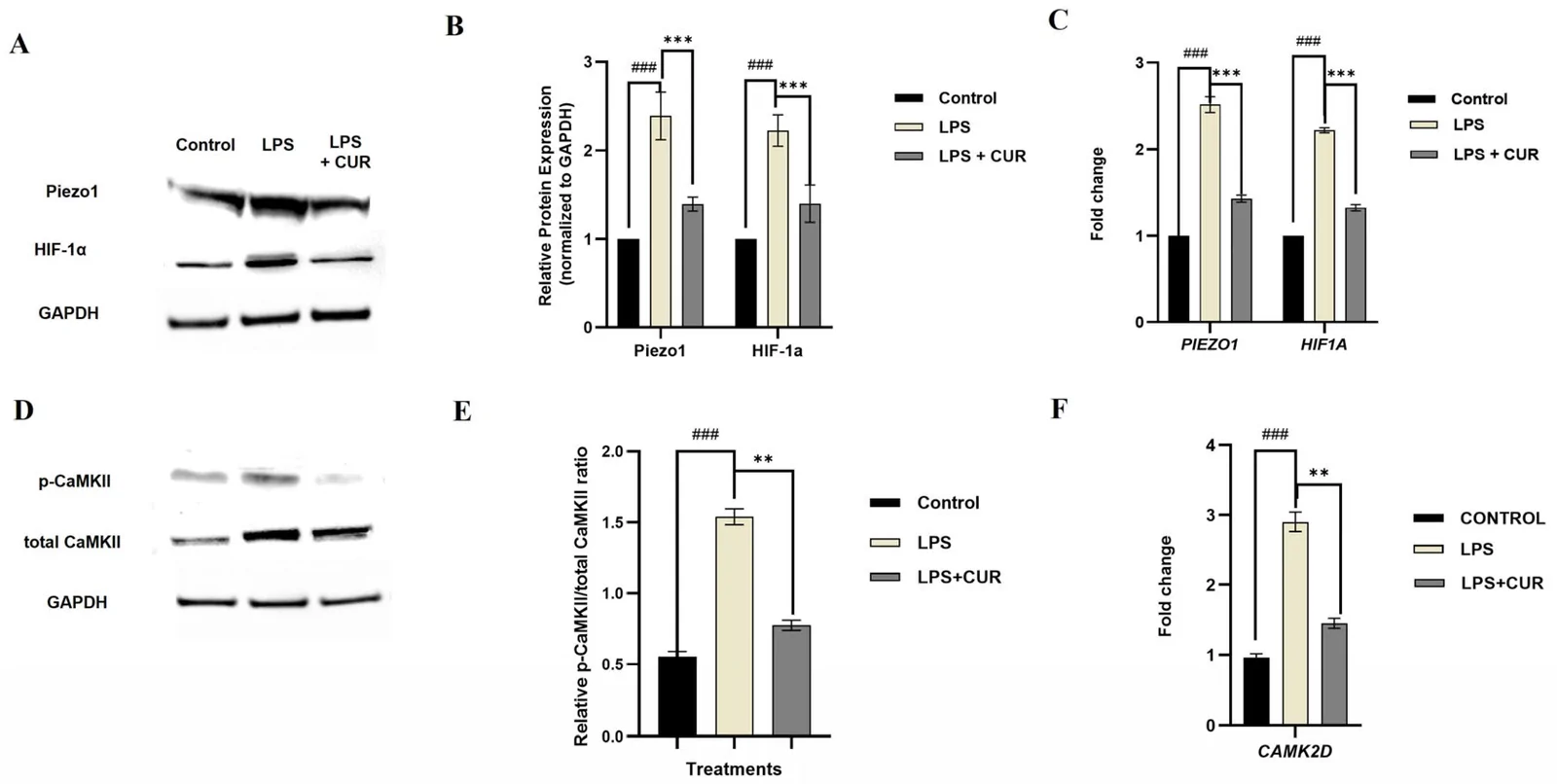
Analysis of Piezo1 and calcium-responsive molecular marker expression in scaffold-free chondrogenic co-culture organoids. (**A**) Representative Western blot images showing Piezo1, hypoxia-inducible factor-1 alpha (HIF-1α), and glyceraldehyde-3-phosphate dehydrogenase (GAPDH) in untreated control, lipopolysaccharide-treated (LPS), and LPS plus curcumin-treated (LPS + CUR; 7.4 µg/mL) organoids. (**B**) Densitometric quantification of Piezo1 and HIF-1α protein abundance normalized to GAPDH. (**C**) Relative expression of human PIEZO1 and HIF1A transcripts, determined by RT-qPCR, normalized to human-specific GAPDH, and calculated using the 2^−ΔΔCq^ method. (**D**) Representative Western blot images showing phosphorylated calcium/calmodulin-dependent protein kinase II (p-CaMKII), total CaMKII, and GAPDH in the indicated experimental groups. (**E**) Densitometric quantification of CaMKII phosphorylation, expressed as the p-CaMKII/total CaMKII ratio. (**F**) Relative expression of human *CAMK2D* transcripts, determined by RT-qPCR, normalized to human-specific *GAPDH*, and calculated using the 2^−ΔΔCq^ method. LPS stimulation was associated with increased Piezo1 and HIF-1α abundance and an increased p-CaMKII/total CaMKII ratio, whereas curcumin treatment was associated with lower levels of these markers relative to the LPS-treated group. Data are presented as mean ± SD from three independent biological replicates (n = 3). Statistical significance was determined using one-way ANOVA followed by Tukey’s multiple-comparisons test. ^###^ *p* < 0.001 versus the untreated control group; ** *p* < 0.01 and *** *p* < 0.001 versus the LPS-treated group.

LPS stimulation increased Piezo1 and HIF-1α protein abundance and increased the p-CaMKII/total CaMKII ratio compared with untreated controls. Curcumin treatment was associated with lower Piezo1 and HIF-1α protein abundance and a lower p-CaMKII/total CaMKII ratio relative to the LPS-treated group. RT-qPCR analysis similarly demonstrated increased *PIEZO1*, *HIF1A*, and *CAMK2D* transcript expression following LPS stimulation, whereas the expression of these transcripts was lower in the curcumin-treated group relative to the LPS-treated group. These findings demonstrate inflammation-associated changes in Piezo1 expression and calcium-responsive molecular markers. Because mechanical stimulation, Piezo1 channel-activity measurements, intracellular calcium imaging, and Piezo1-specific pharmacological or genetic manipulation were not performed, the findings do not establish mechanically activated Piezo1 signaling or Piezo1-dependent causality (Figure 6A–F).

### 3.8. Curcumin Modulates Mitophagy- and Autophagy-Associated Marker Expression

Mitophagy-associated protein expression was evaluated to determine the effects of inflammatory stress on mitochondrial quality control within chondrogenic co-culture organoids. Western blot analysis demonstrated significantly increased expression of PINK1, Parkin, LC3B-II, and p62/SQSTM1 following LPS treatment compared with untreated controls. Increased accumulation of LC3B-II and p62/SQSTM1 was observed following LPS treatment, indicating altered autophagy-associated marker expression and altered mitochondrial quality-control responses under inflammatory conditions.

**Figure 7:**
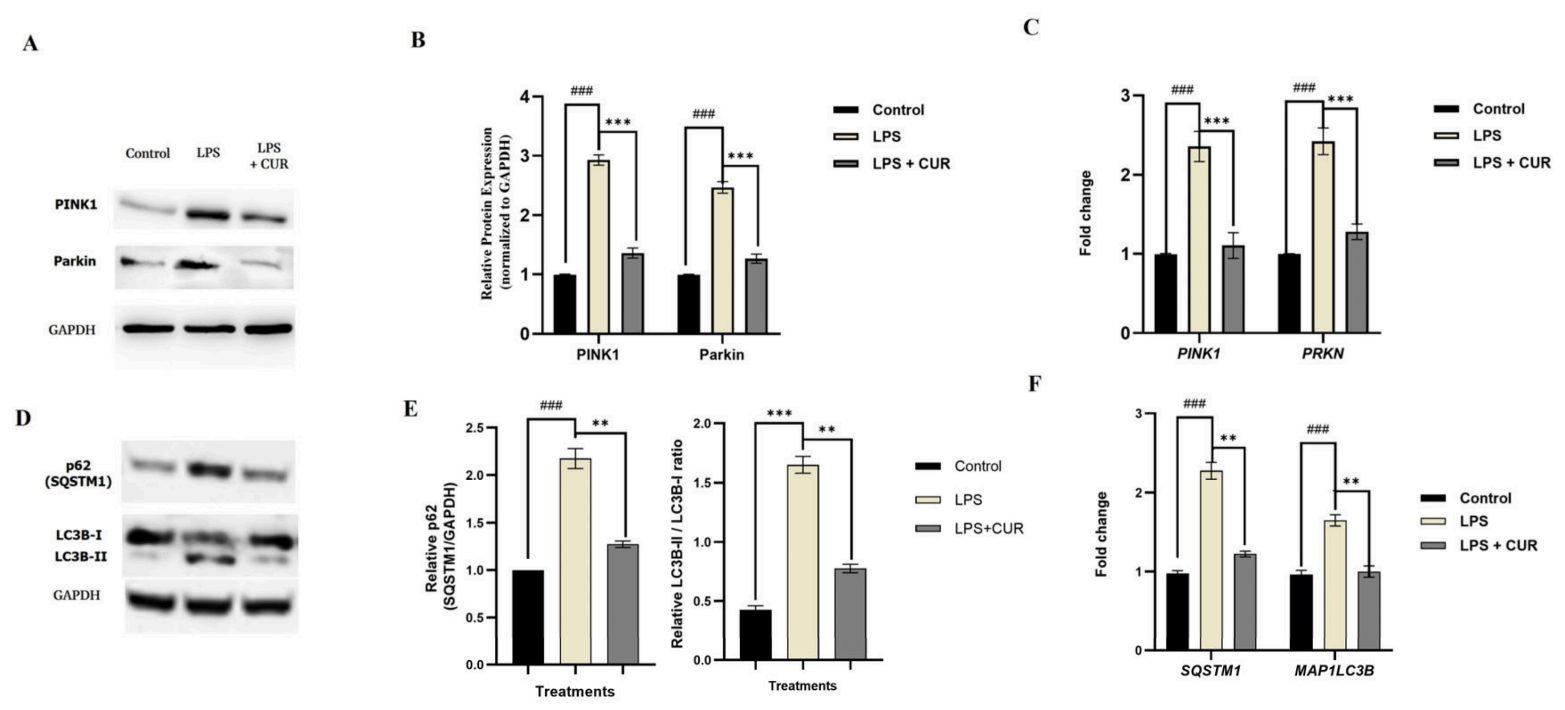
Curcumin modulates mitophagy- and autophagy-associated marker expression in LPS-stimulated chondrogenic co-culture organoids. (**A**) Representative Western blot images showing the expression of the mitophagy-related proteins PINK1, Parkin, and GAPDH in control organoids, LPS-treated organoids, and organoids treated with LPS in the presence of curcumin (7.4 µg/mL). LPS stimulation increased PINK1 and Parkin protein expression, whereas curcumin treatment attenuated their upregulation. (**B**) Densitometric quantification of PINK1 and Parkin protein expression normalized to GAPDH. Protein levels were significantly elevated following LPS exposure and were reduced upon curcumin treatment, indicating modulation of mitochondrial quality-control pathways. (**C**) Relative mRNA expression levels of human *PINK1* and *PRKN*, determined by RT-qPCR, normalized to human-specific *GAPDH*, and calculated using the 2^−ΔΔCq^ method. (**D**) Representative Western blot images showing the expression of p62/SQSTM1, LC3B-I, LC3B-II, and GAPDH in control, LPS-treated, and LPS + curcumin-treated organoids. (**E**) Quantitative densitometric analysis of p62/SQSTM1 protein expression and the LC3B-II/LC3B-I ratio, normalized to GAPDH. LPS treatment resulted in p62 accumulation and an increased LC3B-II/LC3B-I ratio, indicating altered autophagy-associated processing. Curcumin treatment attenuated the observed changes in p62 abundance and the LC3B-II/LC3B-I ratio. (**F**) Relative mRNA expression levels of human *SQSTM1* and *MAP1LC3B*, determined by RT-qPCR, normalized to human-specific *GAPDH*, and calculated using the 2^−ΔΔCq^ method. LPS significantly increased the expression of autophagy-associated genes, whereas curcumin attenuated these changes, consistent with partial modulation of autophagy-associated markers. Data are presented as mean ± SD (n = 3 independent experiments). Statistical significance was determined using one-way ANOVA followed by Tukey’s multiple comparisons test. ^###^ *p* < 0.001 versus Control; ** *p* < 0.01, *** *p* < 0.001 versus LPS-treated group.

Curcumin treatment reduced the expression of PINK1, Parkin, LC3B-II, and p62 relative to the LPS group, suggesting partial modulation of mitochondrial quality-control and autophagy-associated responses. RT-qPCR analysis demonstrated similar expression trends for mitophagy-related genes, consistent with the protein expression findings in regulating mitochondrial quality control pathways (Figure 7A–F).

### 3.9. Curcumin Attenuates Inflammasome Activation and Extracellular Matrix Degradation

Inflammasome activation and ECM-associated proteins were analyzed to evaluate inflammatory and catabolic responses within chondrogenic co-culture organoids. LPS stimulation significantly increased expression of NLRP3 and cleaved Caspase-1 p20 compared with untreated controls, suggesting activation of inflammasome-associated inflammatory responses.

Simultaneously, LPS-treated organoids demonstrated marked reduction of the cartilage matrix protein COL2A1 together with increased expression of the catabolic enzyme MMP13, consistent with a catabolic ECM response.

**Figure 8:**
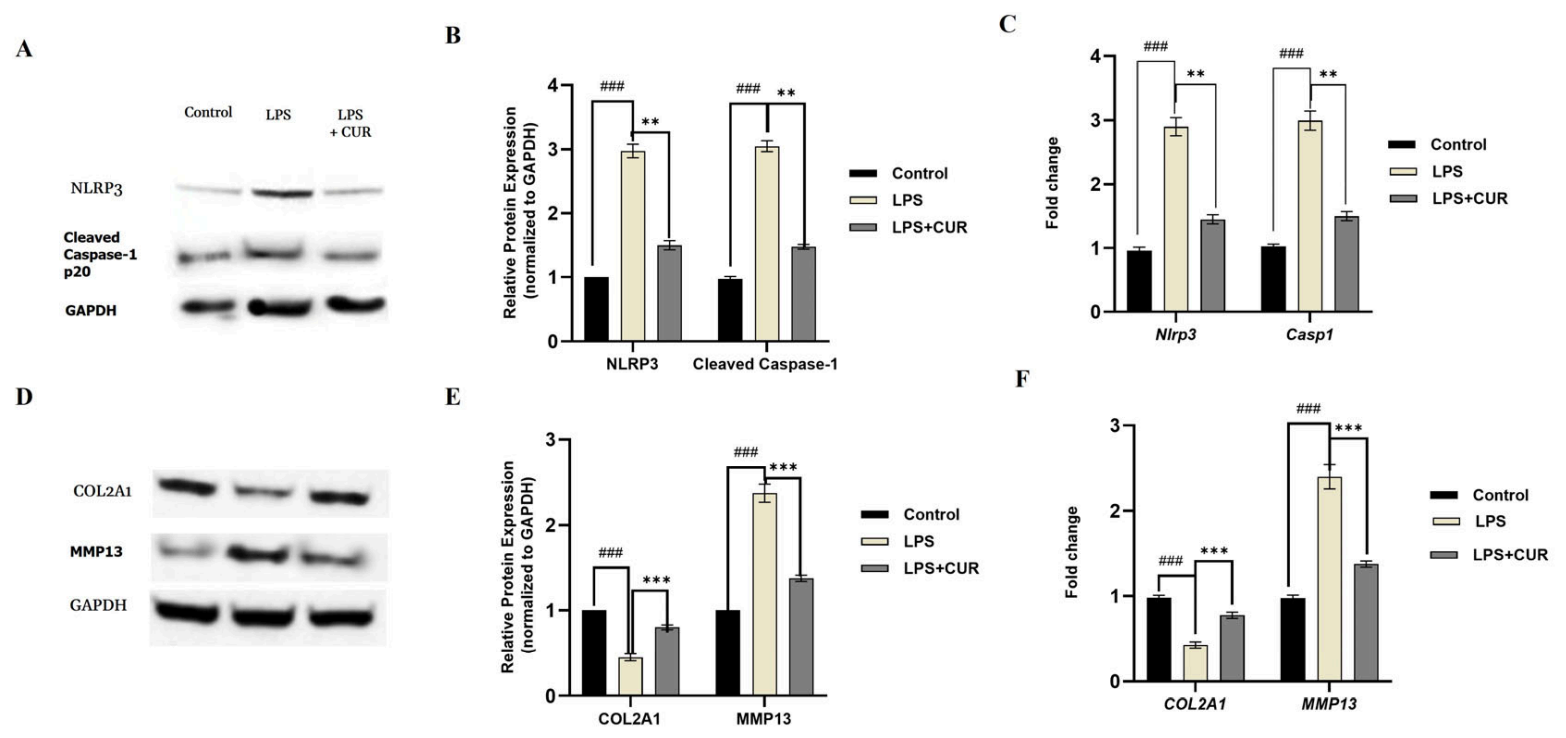
Curcumin attenuates inflammasome activation and preserves extracellular matrix homeostasis in LPS-stimulated chondrogenic co-culture organoids. (**A**) Representative Western blot images showing the protein expression of NLRP3, cleaved Caspase-1 p20, and GAPDH in untreated control organoids, LPS-treated organoids, and organoids treated with LPS in the presence of curcumin (7.4 µg/mL). (**B**) Densitometric analysis of NLRP3 and cleaved Caspase-1 p20 protein expression normalized to GAPDH. LPS stimulation markedly increased the expression of inflammasome-associated proteins compared with the control group, whereas curcumin treatment significantly reduced their expression. (**C**) Relative expression levels of murine *Nlrp3* and *Casp1* transcripts, determined by RT-qPCR, normalized to mouse-specific *Gapdh*, and calculated using the 2^−ΔΔCq^ method. LPS significantly upregulated inflammasome-related gene expression, while curcumin treatment attenuated this response. (**D**) Representative Western blot images showing the expression of extracellular matrix-associated proteins COL2A1, MMP13, and GAPDH in control, LPS-treated, and LPS + curcumin-treated organoids. (**E**) Densitometric quantification of COL2A1 and MMP13 protein expression normalized to GAPDH. Exposure to LPS reduced COL2A1 expression and increased MMP13 expression, indicating matrix degradation. Curcumin treatment partially restored COL2A1 levels and suppressed MMP13 expression. (**F**) Relative expression levels of human *COL2A1* and *MMP13* transcripts, determined by RT-qPCR, normalized to human-specific *GAPDH*, and calculated using the 2^−ΔΔCq^ method. Curcumin treatment mitigated the LPS-induced reduction in COL2A1 expression and significantly decreased MMP13 expression, consistent with preservation of extracellular matrix integrity. Data are presented as mean ± SD from three independent biological replicates (n = 3). Statistical significance was determined using one-way ANOVA followed by Tukey’s multiple-comparisons test. Hash symbols indicate comparisons with the untreated control group: ^###^ *p* < 0.001 versus Control. Asterisks indicate comparisons with the LPS-treated group: ** *p* < 0.01, *** *p* < 0.001.

Curcumin treatment significantly reduced NLRP3 and cleaved Caspase-1 expression while partially preserving COL2A1 expression and reducing MMP13 expression relative to the LPS group. These findings suggest that curcumin attenuates LPS-associated inflammatory and matrix-degrading responses within scaffold-free co-culture organoids. (Figure 8A–F).

## 4. Discussion

The present study established a scaffold-free 3D chondrogenic–osteoclast-like co-culture organoid system for investigating inflammation-associated Piezo1 expression, mitophagy-related markers, and matrix-associated responses under LPS-induced inflammatory conditions. The organoids demonstrated cartilage-like extracellular matrix deposition and chondrogenic marker expression, together with inflammatory activation and alterations in mitophagy- and autophagy-associated markers following LPS stimulation. Curcumin treatment attenuated several LPS-associated inflammatory and catabolic molecular changes and partially preserved cartilage-associated matrix markers. These findings demonstrate concurrent molecular associations but do not establish mechanically activated Piezo1 signaling or Piezo1-dependent causality, mitophagy-associated pathways, inflammasome activation, and ECM degradation.

The present study identifies associations among Piezo1-related molecular changes, mitophagy-associated markers, and inflammasome-associated inflammatory responses within a scaffold-free 3D co-culture organoid system. Compared with conventional monolayer culture, the scaffold-free organoid provided a three-dimensional platform supporting cell–cell interactions and endogenous extracellular matrix organization. Scaffold-free organoids are particularly advantageous because they allow spontaneous cellular self-assembly and formation of tissue-like architecture without interference from artificial biomaterials [45,46]. In the present study, successful formation of compact spherical organoids together with positive Alcian Blue, Toluidine Blue, and Safranin O staining confirmed the establishment of a cartilage-like ECM environment. Strong SOX9 and COL2A1 expression further supported successful chondrogenic maturation within the organoid model.

The continuing limitations of available OA treatments emphasize the need for improved preclinical platforms for therapeutic discovery and evaluation [47]. Organoid-based models are increasingly being applied to disease modelling and therapeutic discovery in cartilage and OA research. Previous studies using human cartilage organoids have demonstrated their utility for pharmacological screening and identification of pathways associated with cartilage regeneration. Engineered human OA cartilage organoids have also been developed to reproduce selected disease-associated phenotypes and evaluate responses to candidate therapeutic interventions. In addition, recent studies incorporating bioactive compounds into cartilage-organoid platforms have demonstrated the potential of combining three-dimensional disease models with pharmacological and immunomodulatory approaches. Collectively, these findings support the emerging role of organoid systems in preclinical OA drug evaluation while highlighting the need for standardized cell sources, disease-induction protocols, mechanical stimulation, and subsequent in vivo validation. The present scaffold-free co-culture system contributes to this developing field by incorporating hBMSC-derived chondrocyte-like cells and RAW264.7-derived osteoclast-like cells and evaluating selected bioactive compounds under controlled LPS-induced inflammatory conditions.

OA is no longer considered solely a degenerative cartilage disorder, but rather a multifactorial disease involving inflammation, mitochondrial dysfunction, abnormal mechanotransduction, and metabolic dysregulation [48,49]. Mechanical stress-associated signaling pathways have recently gained attention in OA pathogenesis, particularly Piezo1-mediated calcium influx signaling. Piezo1 is a mechanosensitive ion channel that responds to abnormal mechanical and inflammatory stimuli and has been associated with cartilage degeneration and chondrocyte dysfunction [7,50]. Although Piezo1 is widely recognized as a mechanosensitive ion channel, no mechanical stimulation was applied in the present study. Therefore, increased Piezo1 abundance following LPS exposure should be interpreted as an inflammation-associated expression response rather than evidence of mechanically activated Piezo1 signaling. The accompanying increase in CaMKII phosphorylation demonstrates a concurrent change in a calcium-responsive signaling marker but does not establish that Piezo1-mediated calcium influx was responsible, because intracellular calcium and Piezo1 channel activity were not directly measured. Moreover, CaMKII can be activated through multiple Piezo1-independent pathways. Direct evaluation using controlled mechanical loading, calcium imaging, electrophysiology, the Piezo1 agonist Yoda1, the mechanosensitive ion-channel inhibitor GsMTx4, or Piezo1-specific genetic modulation will be required to establish the functional and causal involvement of Piezo1.

Increased HIF-1α expression was observed following LPS stimulation and may reflect metabolic adaptation to inflammatory and hypoxic stress within the organoid microenvironment. Previous studies have shown that HIF-1α contributes to altered glycolytic metabolism, mitochondrial dysfunction, and inflammatory signaling in osteoarthritic cartilage [51,52]. However, glycolytic activity was not directly evaluated using glucose uptake, lactate production, glycolytic enzyme expression, or extracellular acidification measurements. Therefore, the observed HIF-1α changes should be interpreted as metabolism-associated stress signaling rather than direct evidence of glycolytic or immunometabolic reprogramming.

The inflammatory induction mechanism represents an important limitation of the present study. Clinical OA is predominantly associated with sterile, low-grade, and chronic inflammation involving damage-associated molecular patterns (DAMPs), inflammatory cytokines, cellular senescence, matrix degradation products, and mechanical stress. LPS, in contrast, is an exogenous bacterial TLR4 agonist that produces an acute inflammatory response. Therefore, the present system should be interpreted as an LPS-induced TLR4-mediated inflammatory pathway model exhibiting selected OA-relevant inflammatory, catabolic, and matrix-associated changes rather than as a model reproducing the complete pathophysiology of OA. This distinction is particularly important because the RAW264.7-derived component may retain strong responsiveness to LPS following M-CSF/RANKL-mediated osteoclast-like differentiation. Consequently, some inflammatory signals may originate predominantly from the murine RAW264.7-derived cells. The use of species-specific RT-qPCR assays allowed transcriptional responses from the human and murine components to be evaluated separately; however, whole-organoid Western blotting could not determine the cellular origin of the measured proteins.

Mitochondrial dysfunction and altered mitophagy-associated responses are increasingly recognized as important contributors to OA progression. Physiological mitophagy maintains mitochondrial quality by eliminating damaged mitochondria and preventing excessive reactive oxygen species accumulation [53,54]. However, altered mitophagy- and autophagy-associated responses may contribute to persistent inflammatory activation and chondrocyte degeneration. In the present study, LPS-treated organoids demonstrated increased PINK1, Parkin, LC3B-II, and p62 expression. Although elevated PINK1 and Parkin expression may initially reflect activation of mitochondrial quality-control mechanisms, the concurrent increases in p62 and LC3B-II indicate altered autophagy-associated marker expression but cannot independently distinguish enhanced autophagosome formation from impaired lysosomal degradation under inflammatory conditions. Similar alterations in autophagy-associated markers have been reported in OA-related experimental systems [44,54].

However, the present measurements represent static assessments of mitophagy- and autophagy-associated markers. Without lysosomal inhibition or a flux-specific reporter, changes in LC3B-II and p62 cannot distinguish increased autophagosome formation from impaired lysosomal degradation. Similarly, changes in PINK1 and Parkin expression alone do not directly demonstrate mitochondrial damage or mitochondrial clearance. Therefore, these findings indicate alterations in mitophagy- and autophagy-associated marker expression rather than confirmed impairment or restoration of mitophagic flux.

Inflammasome activation is another critical mechanism implicated in OA-associated inflammation. The NLRP3 inflammasome contributes to maturation of pro-inflammatory cytokines and amplification of cartilage catabolism during OA progression [54]. Increased expression of NLRP3 and cleaved Caspase-1 observed in LPS-treated organoids in the present study is consistent with previous findings demonstrating inflammasome activation in response to mitochondrial stress and inflammatory stimulation. Simultaneously, reduced COL2A1 expression together with elevated MMP13 expression indicated progressive ECM degradation and cartilage catabolism within the organoid system. MMP13 is a major collagen-degrading enzyme associated with OA progression and cartilage destruction [55].

Among the tested compounds, curcumin produced the greatest improvement in organoid viability relative to the LPS-treated group. Curcumin is a naturally derived polyphenolic compound with reported anti-inflammatory, antioxidant, and mitochondrial protective properties [56,57,58,59]. In the present study, curcumin treatment reduced Piezo1, HIF-1α, and p-CaMKII expression and altered mitophagy-associated marker expression relative to the LPS-treated group. Curcumin additionally suppressed NLRP3 inflammasome activation, reduced MMP13 expression, and partially preserved COL2A1 expression. These findings are generally consistent with previous reports, demonstrating that curcumin attenuates inflammatory signaling, oxidative stress, and cartilage degeneration in experimental OA models [59,60]. Because curcumin is a pleiotropic phytochemical capable of modulating multiple inflammatory, oxidative stress, and survival pathways [58], the observed protective effects cannot be attributed exclusively to modulation of Piezo1-associated signaling. Additional studies employing selective Piezo1 agonists, antagonists, or genetic modulation will be required to establish a direct mechanistic relationship. The findings further support the therapeutic potential of naturally derived bioactive compounds and nutraceutical agents as adjunct strategies for modulating inflammatory and mitochondrial stress-associated pathways in OA. Integration of advanced organoid systems with naturally occurring compounds may provide a controlled exploratory platform for evaluating candidate interventions before validation in fully human, mechanically stimulated, and patient-derived systems. These findings support further investigation of Piezo1-associated pathways in OA, using direct pharmacological and genetic validation [61]. Nevertheless, the clinical translation of curcumin remains challenging because of its limited aqueous solubility, poor oral bioavailability, and rapid systemic metabolism [57]. Future studies should determine whether therapeutically relevant curcumin exposure can be achieved in vivo and evaluate optimized delivery approaches, including nanoparticle-based, liposomal, biomaterial-assisted, or local intra-articular formulations.

**Figure 9:**
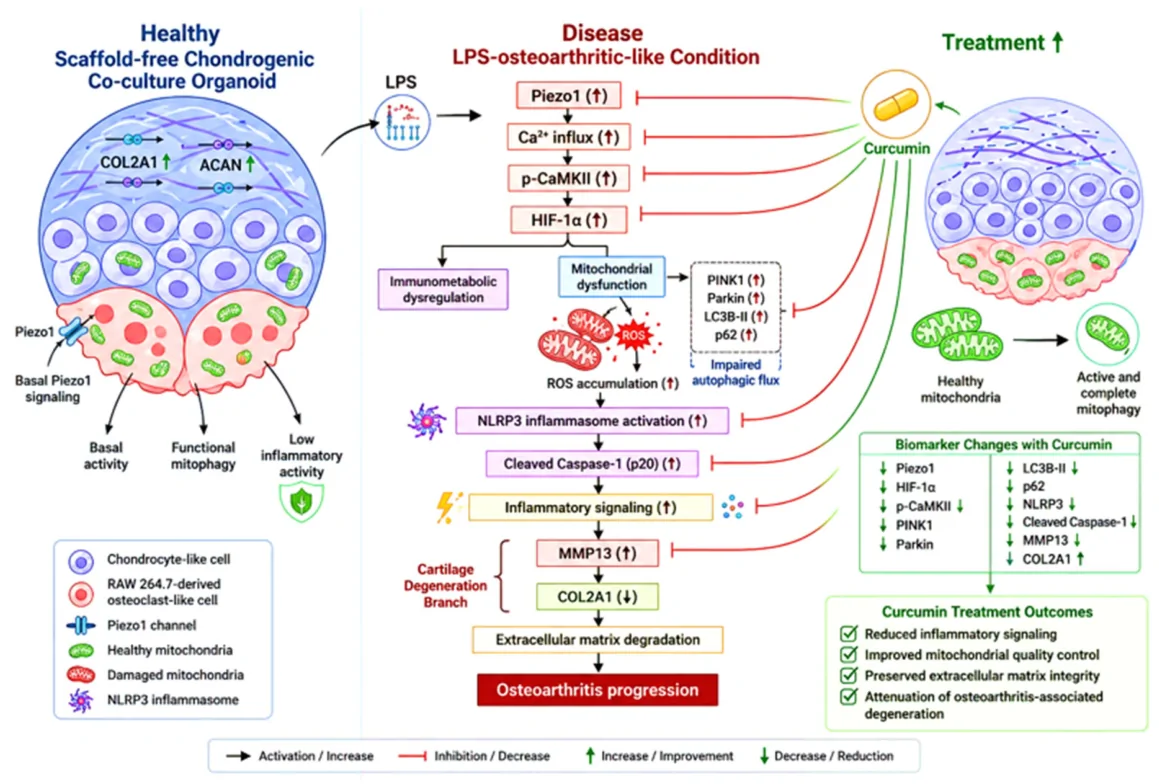
Conceptual summary of molecular changes observed in the LPS-stimulated organoid system. LPS exposure was associated with increased Piezo1 and HIF-1α expression, enhanced CaMKII phosphorylation, and altered expression of PINK1, Parkin, LC3B, p62/SQSTM1, NLRP3, COL2A1, and MMP13. Curcumin treatment attenuated several of these LPS-associated molecular alterations. The arrows and pathway arrangement provide a hypothesis-generating representation of concurrent molecular changes based on the observed expression patterns and relevant literature. These relationships represent proposed associations rather than experimentally established signaling directionality or causality. Functional confirmation will require direct assessment of Piezo1 channel activity, intracellular calcium influx, mitochondrial function, and autophagic/mitophagic flux, together with Piezo1-specific pharmacological or genetic modulation.

A strength of the present study is the integration of a scaffold-free co-culture organoid platform with the evaluation of inflammation-associated Piezo1 expression and mitophagy-related molecular markers. The combined histological and molecular assessments provided complementary characterization of LPS-induced inflammatory, catabolic, and matrix-associated responses and their modulation by curcumin. The proposed molecular associations observed in the LPS-stimulated organoid system are summarized in Figure 9.

This study has several limitations. First, LPS is an exogenous bacterial TLR4 agonist that induces an acute inflammatory response and does not reproduce the sterile, low-grade, chronic, and mechanically influenced pathogenesis of clinical OA. The system should therefore be interpreted as an LPS-induced TLR4-mediated inflammatory model exhibiting selected OA-relevant features rather than as a comprehensive model of OA. Second, no mechanical loading was applied, Piezo1 channel activity and intracellular calcium influx were not measured, and Piezo1-specific pharmacological or genetic interventions were not performed. Consequently, the findings demonstrate inflammation-associated changes in Piezo1 expression and related molecular markers but do not establish mechanotransduction, Piezo1 activation, or Piezo1-dependent causality. Third, hBMSC-derived chondrocyte-like cells are not equivalent to primary adult articular chondrocytes. Although chondrogenic characteristics were supported by cartilage-associated histological staining and SOX9 and COL2A1 expression, differentiation purity was not determined at the single-cell level, and hypertrophic differentiation markers, including COL10A1 and RUNX2, were not assessed. Fourth, although RAW264.7 cells were differentiated using M-CSF and RANKL and osteoclast-like differentiation was supported by the presence of TRAP-positive multinucleated cells, functional bone-resorption activity was not evaluated. The RAW264.7-derived cells may also retain strong intrinsic responsiveness to LPS and may contribute disproportionately to the observed inflammatory response. The xenogeneic composition of the organoid may additionally affect cytokine–receptor compatibility and bidirectional paracrine communication. Although species-specific RT-qPCR assays distinguished transcript-level responses associated with the human and murine components, whole-organoid Western blotting and the absence of corresponding monoculture controls prevented definitive assignment of the measured proteins and other responses to either cell population. During preliminary optimization, THP-1 cells were evaluated as a potential human monocytic source; however, they did not provide a sufficiently viable and reproducible osteoclast-like cell yield for incorporation into the 3D co-culture system [61,62]. RAW264.7 cells were therefore used because of their established and reproducible M-CSF/RANKL-induced osteoclastogenic potential [63]. The organoid also lacks other components of the native joint microenvironment, including synovial cells. Compound-only controls were not included; therefore, direct effects of curcumin, rivaroxaban, and S1P on basal organoid physiology cannot be excluded. Furthermore, mitochondrial function and autophagic/mitophagic flux were not directly assessed, and the effects of curcumin were evaluated in vitro only. Future studies should employ patient-derived, fully human co-culture systems, sterile OA-relevant inflammatory stimuli, chronic exposure paradigms, controlled mechanical loading, monoculture and compound-only controls, direct Piezo1 functional measurements, bone-resorption assays, and mitochondrial-functional and autophagic-flux assessments.

## 5. Conclusions

This study established an LPS-induced scaffold-free chondrogenic–osteoclast-like co-culture organoid system exhibiting selected features of OA-relevant inflammation and cartilage matrix dysregulation. LPS exposure was associated with increased Piezo1 expression and concurrent alterations in inflammatory, catabolic, mitophagy-related, and autophagy-related markers. Curcumin attenuated several of these molecular changes and partially preserved cartilage-associated matrix markers. However, the findings do not establish mechanically activated Piezo1 signaling, identify Piezo1 as a direct molecular target of curcumin, or demonstrate restoration of mitophagic flux. The model should therefore be considered an exploratory TLR4-mediated inflammatory platform for investigating molecular associations and candidate interventions, with further validation required in mechanically stimulated, fully human, and patient-derived OA systems.

## Data Availability

The raw data (including image analysis spreadsheets and raw Western blot images with molecular weight markers) supporting the conclusions of this article will be made available by the corresponding author on request.

## Supplementary Materials

The following supporting information can be downloaded at: https://www.mdpi.com/article/10.3390/cells15161482/s1, Figure S1: Original uncropped Western blot images corresponding to Figure 6, Figure 7 and Figure 8; Figure S2: Validation of RAW264.7-derived osteoclast-like cell differentiation. (A) RAW264.7 cells at baseline, displaying a predominantly small, round morphology under phase-contrast microscopy. (B) Cells following 5 days of stimulation with M-CSF (50 ng/mL) and RANKL (50 ng/mL), showing increased cell size and spreading consistent with osteoclastogenic differentiation. (C) Enlarged multinucleated cells exhibiting osteoclast-like morphology; arrows indicate representative multinucleated cells. (D) Tartrate-resistant acid phosphatase (TRAP) staining demonstrating TRAP-positive osteoclast-like cells, with prominent cytoplasmic staining in multinucleated cells; arrows indicate representative TRAP-positive multinucleated cells containing three or more nuclei. (E) Undifferentiated RAW264.7 cells cultured without MCSF and RANKL and subjected to TRAP staining, showing minimal TRAP-positive staining and an absence of multinucleated cell formation. Representative images from the differentiation-validation experiment areshown. Scale bars = 100 μm (A–E); Table S1: Primary antibodies used for Western blot analysis.
