# Supplementary material for: Inflammation-Associated Changes in Piezo1 Expression, Mitophagy-Related Markers, and Matrix Dysregulation in an LPS-Stimulated Co-Culture Organoid System
Source: Cells. 2026 Aug 18;15(16):1482. doi: 10.3390/cells15161482 (PMC13511527; doi:10.3390/cells15161482)
Supplement: Supplementary file 1 [file cells-15-01482-s001.zip › cells-4459367-supplementary.pdf]

Supplementary Figure S1. Original uncropped Western blot images corresponding to Figures 6–8.

Original, uncropped Western blot images used for the preparation of **Figures 6–8** are provided. The highlighted regions correspond to the lanes presented in the main manuscript. Protein bands were detected using chemiluminescence, and the corresponding molecular weight markers are shown where available. These images represent the original, unprocessed blots used for densitometric analysis.

Figure 6.

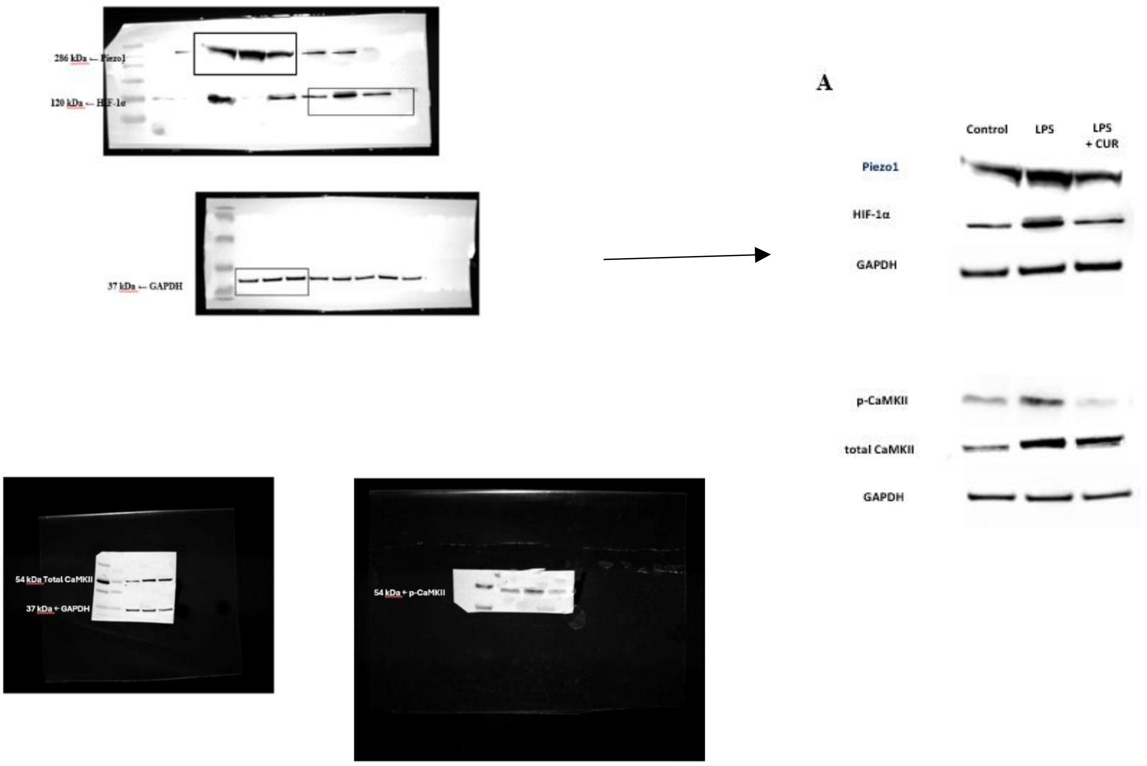

Dubai Health - Internal Use

Figure 7.

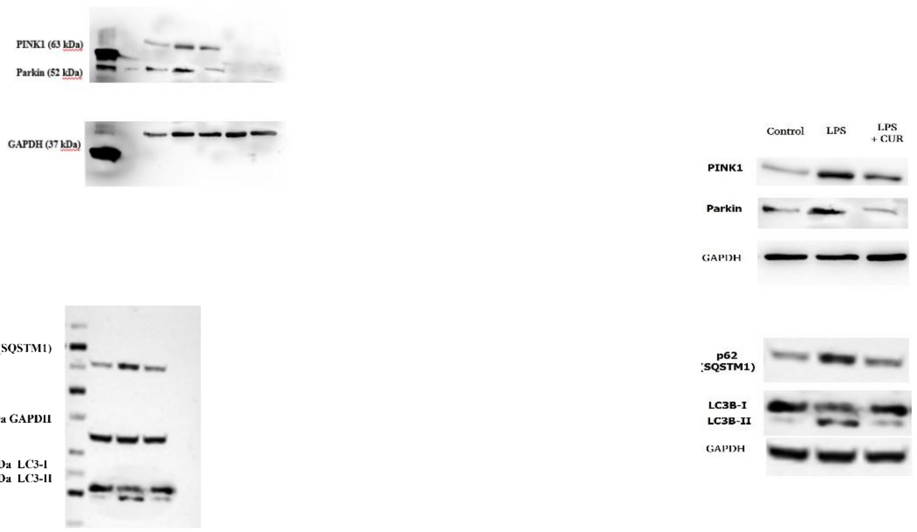

Figure 8

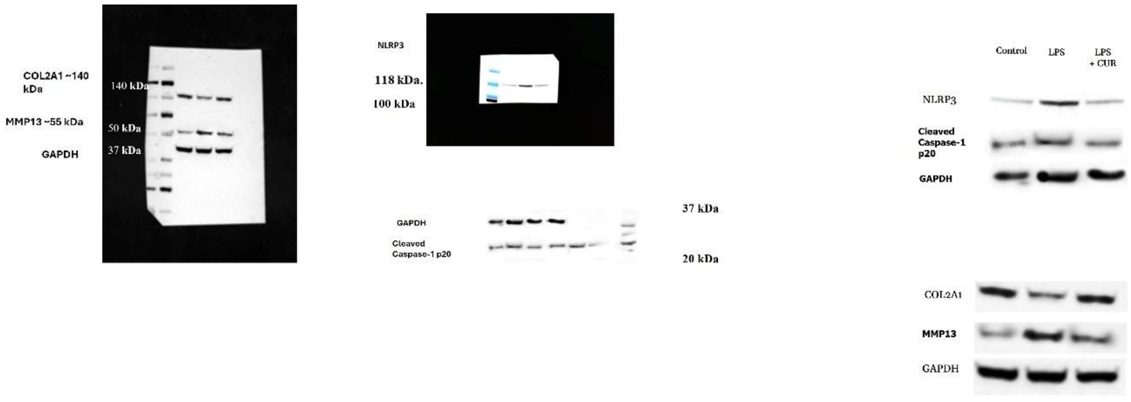

Supplementary Figure S2. Validation of RAW264.7-derived osteoclast-like cell differentiation. (A) RAW264.7 cells at baseline, displaying a predominantly small, round morphology under phase-contrast microscopy. (B) Cells following 5 days of stimulation with M-CSF (50 ng/mL) and RANKL (50 ng/mL), showing increased cell size and spreading consistent with osteoclastogenic differentiation. (C) Enlarged multinucleated cells exhibiting osteoclast-like morphology; arrows indicate representative multinucleated cells. (D) Tartrate-resistant acid phosphatase (TRAP) staining demonstrating TRAP-positive osteoclast-like cells, with prominent cytoplasmic staining in multinucleated cells; arrows indicate representative TRAP-positive multinucleated cells containing three or more nuclei. (E) Undifferentiated RAW264.7 cells cultured without M-CSF and RANKL and subjected to TRAP staining, showing minimal TRAP-positive staining and an absence of multinucleated cell formation. Representative images from the differentiation-validation experiment are shown. Scale bars = 100 µm (A–E).

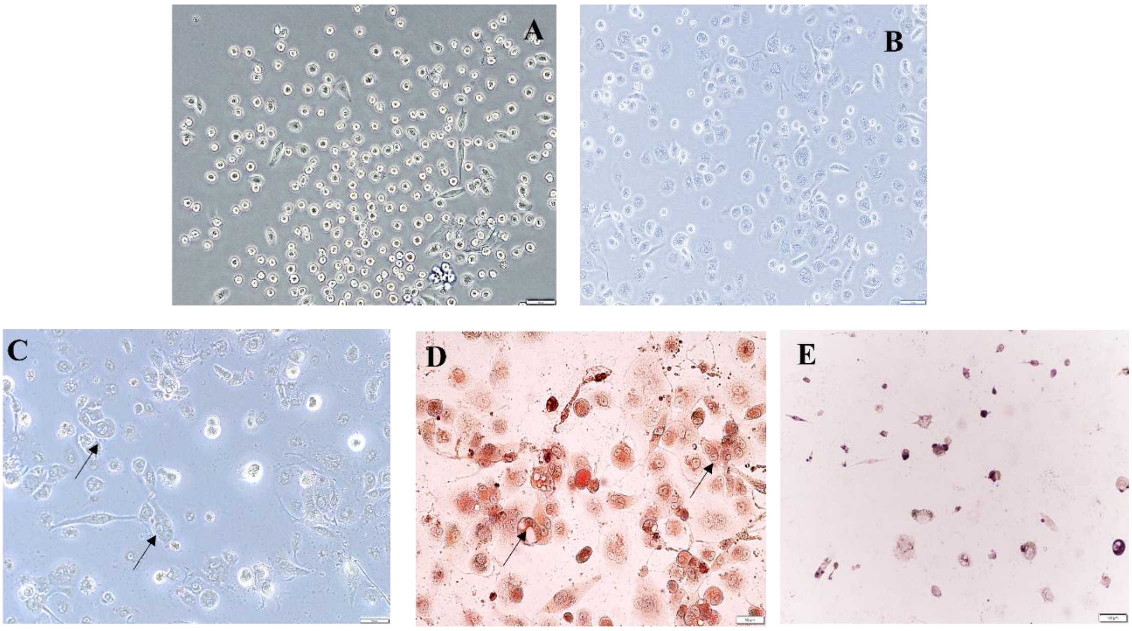

Supplementary Table S1. Primary antibodies used for Western blot analysis

Target Protein	Company	Catalogue Number	Host Species	Dilution	Expected Molecular Weight
Piezo1	Abcam	ab128245	Rabbit	1:1000	~286 kDa
HIF-1α	Abcam	ab179483	Rabbit	1:1000	~120 kDa
Phospho-CaMKII (Thr286)	Cell Signaling Technology	#12716	Rabbit	1:1000	~54 kDa
Total CaMKII	Cell Signaling Technology	#4436	Rabbit	1:1000	~54 kDa
PINK1	Abcam	ab23707	Rabbit	1:1000	~63 kDa
Parkin	Abcam	ab77924	Rabbit	1:1000	~52 kDa
LC3B	Cell Signaling Technology	#3868	Rabbit	1:1000	LC3-I ~16 kDa; LC3-II ~14 kDa
p62/SQSTM1	Abcam	ab56416	Rabbit	1:1000	~62 kDa
NLRP3	Abcam	ab263899	Rabbit	1:1000	~118 kDa

Dubai Health - Internal Use

Target Protein	Company	Catalogue Number	Host Species	Dilution	Expected Molecular Weight
Cleaved Caspase-1 (p20)	Cell Signaling Technology	#89332	Rabbit	1:1000	~20 kDa
COL2A1	Abcam	ab34712	Rabbit	1:1000	~135 kDa
MMP13	Abcam	ab39012	Rabbit	1:1000	~60 kDa
GAPDH	Cell Signaling Technology	#5174	Rabbit	1:5000	~37 kDa

Appropriate horseradish peroxidase (HRP)-conjugated secondary antibodies were used according to the manufacturer's recommendations. Protein bands were visualized using enhanced chemiluminescence (ECL) detection and quantified using ImageJ software.

Dubai Health - Internal Use
